# Evaluation of the antidiarrheal activity of 80% methanol extract and solvent fractions of the leaf of *Bersama abyssinica* fresen (Melianthaceae) in mice

**DOI:** 10.1186/s12906-021-03498-6

**Published:** 2022-01-06

**Authors:** Mihret Ayalew, Azmeraw Bekele, Mestayet Geta Mengistie, Seyfe Asrade Atnafie

**Affiliations:** 1 Department of Pharmacology, Institute of Health, Jimma University, Jimma, Ethiopia; 2 Department of Social and Administrative Pharmacy, Institute of Health, Jimma University, Jimma, Ethiopia; 3 Department of Pharmacology, School of Pharmacy, College of Medicine and Health Science, University of Gondar, Gondar, Ethiopia

**Keywords:** In vivo, Antidiarrheal, *Bersama abyssinica*, Castor oil, Mice

## Abstract

**Introduction:**

The use of traditional medicinal plants in the management of diarrhea has long been practiced in Ethiopia. *B. abyssinica* fresen is one of the plants traditionally used to treat diarrhea whereas an in vivo study had not yet been conducted. Thus, this study aimed to evaluate the antidiarrheal activity of crude extract and solvent fractions of the leaf of *B. abyssinica* in mice.

**Methods:**

Cold maceration within 80% methanol was used to extract the leaf powder and extract of the leaf was fractionated using n-hexane, chloroform, and distilled water. The in vivo antidiarrheal activity of crude extracts and solvent fractions were tested in experimental models of castor oil-induced diarrhea, enteropooling, and antimotility test. Five groups each with 6 mice were used under the three antidiarrheal models. Positive controls were treated with loperamide 3 mg/kg and atropine 5 mg/kg and 2% tween 80 was used in the treatment of negative controls. The extract and solvent fractions were administered at doses of 100, 200, and 400 mg/kg. Time of onset of diarrhea, number and weight of total and wet feces, the percent reduction in the number of wet feces, weight and volume of intestinal contents, and percent inhibition of intestinal motility were recorded. Data were analyzed using SPSS version 20.

**Result:**

Defecation of castor oil-induced diarrheal or loose stools was inhibited (*p* < 0.01 to *p* < 0.001) at 200 mg/kg and 400 mg/kg of crude extract and aqueous fraction. The crude extract and the aqueous fraction at three doses (*p* < 0.01 to *p* < 0.001), the chloroform fraction at 200 mg/kg and 400 mg/kg (*p* < 0.01 to *p* < 0.001), and the n-hexane fraction at 400 mg/kg (*p* < 0.05) reduced intraluminal fluid accumulation compared with the negative control. Castor oil-induced intestinal motility was significantly suppressed with the three-doses of aqueous fraction (*p* < 0.05 to *p* < 0.001), 200 mg/kg and 400 mg/kg of crude extract (*p* < 0.05 to *p* < 0.01), 400 mg/kg of chloroform and n-hexane (*p* < 0.01 to *p* < 0.001) compared with negative control.

**Conclusion:**

The crude extract, aqueous, and chloroform fractions of *B. abyyssinica* leaves have promising anti-diarrheal effects, supporting the plant's traditional use to treat diarrhea.

## Introduction

Diarrhea is defined as three or more bowel movements associated with abnormally loose or liquid stools. The frequency, consistency, and weight of stool are taken into account to identify diarrhea [1]. Diarrhea is forced by osmotic and active secretion, with impaired peristalsis and exudation [2, 3]. There are three diarrheal syndromes; acute diarrhea (250 ml/kg/day of water loss in the stool), bloody diarrhea (with visible blood in the stool), and persistent diarrhea (which lasts two weeks or more). Acute watery diarrhea with dehydration is the leading cause of death from diarrhea [4]. Every year, 1.7 billion cases of diarrhea in children and 525,000 deaths in under-age five children are reported. About half of these diarrheal diseases are reported in South Asia and Africa. Children mortality rates from diarrhea are highest in sub-Saharan Africa [2, 3], while Ethiopia has the fifth-highest diarrhea burden in the world [2, 4].

Diarrhea is both preventable and treatable. However, the treatment of diarrhea is limited by unsafe, ineffective, contraindication of antidiarrheal drugs [1], and/or development of resistance [4, 5]. Medicinal plants (MPs) are the most important and sometimes the only source of diarrhea treatment. Because MPs are culturally accepted, accessible, and inexpensive compared to modern medicine, nearly 80% of the Ethiopian population uses MPs [1].

*Bersama abyssinica (B. abyssinica)* fresen belongs to the Melianthaceae family and the genus *Bersama* [1]. It is a small evergreen tree 12 to 25 m high and its leaves are alternate, impair innately compound and consist of 12 pairs of opposing leaflets with 10–12 pairs of lateral veins. *B. abyssinica* is distributed from Guinea Bissau through the coastal countries of West Africa except for Benin, to the east in Eritrea and Ethiopia, and the south in Angola, Zambia, Zimbabwe, and Mozambique. In Ethiopia, *B. abyssinica* is called Azamir (Amharic), and Lolchissa (Oromeffa) [2].

*B. abyssinica* fresen is rich in various secondary metabolites responsible for the drug's effects in the treatment of various ailments. The stem bark of *B. abyssinica* is rich in phenols, flavonoids, and saponins [1]. Methanolic fraction of leaf, stem bark, and root bark of *B. abyssinica* contained flavonoids, terpenes, steroid, carotenoid, unsaturated, and saturated fatty acids [6]. The methanolic and chloroform extracts of the leaves of the plant are rich in alkaloids, glycosides, and flavonoids, while the methanolic extract of the leaves contained; steroids, phenols, tannins, triterpenes, anthraquinones, polysterols, and coumarin (3). The aqueous extract of the leaves contains phenolic compounds such as; flavonoids and tannins [4].

*B. abyssinica* fresen is traditionally used to treat various ailments. Among various parts of the plant used, the decoction of the leaves is widely used to treat many stomach ailments such as abdominal pain, diarrhea, cholera, intestinal worms, amoebiasis, and dysentery [5]. The stem bark and root bark of *B. abyssinica,* in Ethiopia, is used to treat dysentery, syphilis, and ascariasis [7]. A leaf decoction is used to treat ringworm [8, 9], stomache [10], and diarrhea [11].

Different traditionally claimed activities of *B. abyssinica* fresen leaf extracts including antidiabetics, antioxidants [4], antimalarial [12], antibacterial [3, 13], anthelmintic [14], antiviral [15], and antifungal [16] have been demonstrated *in vitro* and/or *in vivo* studies. Moreover, the aqueous extract from the leaves of *B. abyssinica* had spasmolytic activity on isolated guinea-pig ileum [17]. Nevertheless, the *in vivo* antidiarrheal activity of the plant has not been investigated yet. Hence, this study aimed to evaluate the *in vivo* antidiarrheal activity of the leaves of *B. abyssinica* fressen.

## Methods

### Animals

Swiss albino mice aged between 6 and 8 weeks and weighing between 20 and 35 g breeding in the animal house of the department of pharmacology, the University of Gondar, was used to test for antidiarrhoeal activity. Animals were kept under standard conditions room with 12 h of light and dark cycles. Animals were fed a standard pellet diet and water *ad libitum*. Before the experiment, animals were acclimatized for a week under laboratory conditions. Animals were handled and used per international guidelines for the care and use of laboratory animals [18].

### Plant material collection and authentication

Fresh leaves of *B. abysinica* fresen were collected from Alem Saga, in the South Gondar zone, in the national state of Amhara, and 154 km from the city of Gondar at 38^0^1^’^E of longitude and latitude of 11^0^51^’^N with an elevation of 2706 m on 25 January 2020. It was transported by wrapping it in a plastic bag. The leaves were identified and authenticated by the botanist of the University of Gondar, Mr.Abiyu Eniyew Molla, and registered with voucher No.001MAT/2020 has been deposited in the herbarium of the Department of Biology, University of Gondar, for future reference.

### Plant extraction and solvent fractionation

The collected leaves were washed to remove dust particles and dried under shade at room temperature for a week. The dried leaves were crushed into a coarse powder with a mortar and pestle. The resulting powder was measured on digital balance (EPH 00 Abron Exports) and obtained 1000 g. The 1000 g of powder was cold macerated using three Erlenmeyer conical flasks of equal size, each containing 2 L of 80% methanol (Meru Chem Pvt. Ltd). It was allowed to wait 3 days with occasional shaking. On day 4, the macerate was first filtered through gauze, followed by Whatman No.1 filter paper. The residue in each macerating flask was re-macerated twice using the same amount of 80% methanol used in the first maceration. Meanwhile, the filtrates were combined and concentrated in a rotary evaporator (Yamato Scientific CO. Ltd, Japan) at 40° C. The filtrate concentrate was frozen in a deep freezer (-20° C) overnight. Then, the water content of the frozen filtrate was removed by lyophilization within a vacuum freeze dryer (Lab Freeze Instruments Group, Ltd, Germany) at -50° C and under vacuum pressure (200 mBar). Finally, the dried filtrate (crude extract) was well packed and refrigerated at -4 °C until use.

From the crude extract, 55 g was fractionated using n-hexane (Pentoky organy, India LTD), chloroform (Carlo ERBA S.A.S reagent), and distilled water (Ethiopia Pharmaceutical Factory, Ethiopia). The measured amount was first dissolved in a 1000 ml glass beaker with 250 ml of distilled water, followed by an equal volume of n-hexane. The bulk solution was suspended in a 500 ml separating funnel and left to stand for a few hours under occasional stirring until a clear layer was formed. After two clear layers formed, the n-hexane content was filtered out. The residue was re-macerated twice with the same volume of n-hexane and in the same way as for the first maceration. Then the filtrate fractions were combined and used as the n-hexane fraction (NHF). The 250 ml of chloroform was added to the residue and repeated in the same manner as n-hexane. The chloroform layer was then separated from the residue and used as the chloroform fraction (CF). The final residue was used as an aqueous fraction (AF). NHF and CF were concentrated on a rotary evaporator and dried in a hot oven (Medit Medizin Technik, Germany) at 40 °C and the AF was lyophilized. Finally, the fractions were kept in the refrigerator until the time of use. After all, the yield of both the crude extract and the solvent fraction was calculated according to the following formula [19].

$$\%yield=\frac{weight\;of\;the\;extract}{Weight\;of\;plant\;material}\times100$$

The percentage yield of the crude extract was 17.30%, while AF, CF, and NHF were 63.64%, 13.09%, and 4.18%, respectively.

### Grouping and dosing of the animal

The mice were randomly grouped into five groups, each containing six mice. These groups were designated as negative controls (group I), positive controls (group II), and the remaining three treatment groups. Negative control mice were treated with 2% tween 80 (10 ml/kg). At the same time, positive controls were treated with loperamide (Brawn Laboratories Ltd, India) (3 mg/kg) for castor oil-induced diarrhea, enterpooling models, and atropine sulfate (Reyoung Pharm) (5 mg/kg) for the antimotility testing model. The three treatment groups were treated with doses of 100 mg/kg (group III), 200 mg/kg (group IV), and 400 mg/kg (group V) of crude extracts or solvent fractions. These doses were determined based on previous acute toxicity studies results that showed LD_50_ of the leaf extract was greater than 2000 mg/kg [11, 20]. The doses of crude extracts and solvent fractions were determined from 2000 mg/kg in that one-tenth used as the middle dose. One-half and double of the middle dose are used as the low and high dose, respectively [21].

### Antidiarrheal activity testing

#### Castor oil-induced diarrhea

The antidiarrheal activity of the crude extracts and the solvent fractions was tested according to the method used by Awouters et al. [22]. The mice were fasted for 18 h for food but had free access to water. The fasted mice were randomly grouped and treated as mentioned in the grouping and dosing sections. After 1 h of dosing, 0.5 ml of castor oil (CO) (Jordan Amman Pharmaceutical Industry CO) was administered orally. Then each mouse was placed in a separate cage lined with transparent paper. The transparent paper was changed every 1 h for 4 h of observation. During the observation period, time of onset of diarrhea (time of first diarrhea following castor oil administration), number and weight of total and wet feces were measured. All, 100% of the feces of the negative control group were assumed to be wet feces (diarrhea). The percentage of diarrhea inhibition was calculated according to the following formula [23].

$$\%ID=\frac{Mean\;number\;of\;(WFNC-WFT)}{Mean\;number\;of\;WFNC}\times100$$

$$\%ITFO=\frac{Mean\;number\;of\;(TFC-TFT)}{Mean\;number\;of\;TFC}\times100$$

*Where, ID* = *inhibition of diarrhea, WFNC* = *wet feces in the negative control group, WFT* = *wet feces in the test group, ITFO* = *Inhibition of total fecal output, TFC* = *total feces of control, and TFT* = *total feces of treated.*

#### Castor oil-induced enteropooling

The activity of crude extracts and solvent fractions in reducing intestinal secretion induced by castor oil was determined according to the method applied by Chitme et al. [24]. The mice were fasted for 18 h for food but had free access to water. Then the mice were randomly grouped and treated as described in the grouping and dosing section. One hour after oral administration, 0.5 ml of CO was administered orally to each mouse. After waiting for an hour, the animals were sacrificed by cervical dislocation, followed by laparotomy with a surgical blade. The small intestine was then cut from the pylorus and cecum and ligated at both ends. Intestinal weight was measured before and after removing bowel contents and the difference was calculated. The intestinal content was removed by milking into the calibrated cylinder for volume measurement. Finally, the percentage reduction in weight and volume of the intestinal content relative to the negative control was measured using the following formula.

$$\%RWI=\frac{MW\;(g)\;(negative\;control\hspace{0.17em}-\hspace{0.17em}treated\;group)}{MW\;(g)\;in\;the\;negative\;control}\times100$$

$$\%RVIC=\frac{MVIC\;(ml)\;(negative\;control\hspace{0.17em}-\hspace{0.17em}treated\;group)}{MVIC\;(ml)\;in\;the\;negative\;control}\times100$$

*Where, %RWI = % reduction in weight of intestinal content, %RVIC = % reduction in the volume of intestinal content, MW = mean weight of intestine, MVIC = mean volume of intestinal content*

#### Antimotility test

The antimotility activity of the crude extract and the solvent fraction was evaluated according to the method described by Teferi et al. [25]. The mice fasted for 18 h for food and free access to water. The fasted animals were randomly grouped and treated as indicated in the sections on grouping and dosing. After 30 min of dosing, 0.5 ml of CO was administered and waited a further 30 min to administer 0.5 ml of marker charcoal (Acuro Organics Ltd, New Delhi) suspension in distilled water using oral gavage. After 30 min of charcoal meal administration, the animals were sacrificed by cervical dislocation, followed by the opening of the abdominal cavity and dissection of the small intestine from the junction of the pylorus and the cecum. The total length of the isolated intestine was measured with a ruler, and the distance the charcoal moves (from the end of the pylorus to the last charcoal spot) was measured and expressed by the peristaltic index (PI), thus determining the percent inhibition of motility using of the formula below.

$$PI=\frac{Distance\;traveled\;by\;the\;charcoal\;marker}{The\;total\;length\;of\;the\;small\;intestine}\times100$$

$$\%Inhibition\;of\;motility=\frac{PI\;(negative\;control-test)\hspace{0.17em}}{PI\;negative\;control}\times100$$

#### *In vivo* antidiarrheal index

*In vivo* antidiarrheal index (ADI) is the measure of the antidiarrheal effectiveness of the plant. It is mathematically calculated based on the formula given below [26].

$$In\mathit\;vivo\mathit\;ADI\mathit=\sqrt[\mathit3]{Dfreq\;\mathit\times\mathit\;Gmeq\mathit\times\mathit\;P\;freq}$$

$$Dfreq=\frac{mean\;onset\;of\;diarrhea\;in\;(treated\;group\hspace{0.17em}-\hspace{0.17em}negative\;control\;group)}{mean\;on\;set\;of\;diarrhea\;in\;the\;negative\;control\;group}\times100$$

*Where, Dfreq is the delay in defecation time or diarrhea onset, Gmeq is the charcoal meal travel reduction (as % of the negative control) from the gastrointestinal motility test model, and Pfreq is the reduction in the number of wet stools (as % of the negative control) from the castor oil-induced diarrhea model.*

### Statistical analysis

The results were presented as the mean ± standard error of the mean (mean ± SEM). Data analysis was performed by one-way ANOVA followed by Tukey's post hoc test using SPSS version 23 statistical software, with a 95% confidence interval. A *p*-value < 0.05 is considered statistically significant.

## Results

### Castor oil-induced diarrhea

Crude plant extracts at doses of 200 mg/kg and 400 mg/kg delayed the onset of diarrhea and significantly reduced the weight of wet feces (*p* < 0.001) compared to the negative control. The 100 mg/kg crude extract also significantly reduced wet feces weight (*p* < 0.05) compared to the negative control. The crude extract at 400 mg/kg significantly delayed the time to onset of diarrhea (*p* < 0.001) compared to its 100 mg/kg. AF at 400 mg/kg delayed the onset of diarrhea and significantly reduced whole, wet feces weight (*p* < 0.001) compared to negative controls. While at a dose of 200 mg/kg, it delayed the onset of diarrhea and significantly decreased the number and weight of wet feces (*p* < 0.01) compared to the negative control. CF at 400 mg/kg delayed the onset of diarrhea and significantly reduced total and wet feces weight (*p* < 0.001) compared to negative controls. AF and CF at 400 mg/kg significantly delayed the onset of diarrhea compared to their 100 mg/kg and 200 mg/kg and were comparable to the effects of loperamide. NHF at 400 mg/kg (*p* < 0.05) reduced the total feces weight compared to the negative control. The crude extract, AF, and CF at 400 mg/kg inhibited diarrhea above 50% (Table 1).

**Table 1:** Effects of crude extract and solvent fractions of the leaf of *B. abyssinica* on castor oil-induced diarrhea

Types of treatment	OD (min)	TNF	NWF	WTF (g)	WWF(g)	% ITFO	% ID
NC	64.50 ± 2.17	9.83 ± 0.70	7.50 ± 0.56	0.62 ± 0.02	0.50 ± 0.02	––	–––-
Loperamide (3 mg/kg)	118.5 ± 4.12	5.83 ± 0.70	2.50 ± 0.42	0.31 ± 0.03	0.15 ± 0.02	40.69	66.67
Crude extract (mg/kg)							
100	76.00 ± 2.31^b3e3^	9.50 ± 0.76^b1^	6.33 ± 0.49 ^b3e1^	0.50 ± 0.53 ^a1b2^	0.37 ± 0.02 ^a2b3d1e2^	3.36	15.6
200	90.67 ± 4.40^a3b3^	7.17 ± 0.83	4.5 ± 0.43 ^a2b1^	0.35 ± 0.03^a3^	0.21 ± 0.0^a3c1^	27.06	40
400	102.33 ± 5.1^a3b1c3^	6.67 ± 0.71^a1^	2.83 ± 0.31^a3c1^	0.34 ± 0.01^a3^	0.16 ± 0.02^a3c2^	32.15	62.67
AF(mg/kg)							
100	80.83 ± 3.20 ^a1b3e2^	9.50 ± 0.85^b1^	6.50 ± 0.50 ^b3e1^	0.59 ± 0.02 ^b3e3^	0.43 ± 0.03^b3e3^	3.36	13.33
200	87.00 ± 2.13^a2b3e1^	8.00 ± 0.82	4.50 ± 0.62^a2^	0.55 ± 0.02 ^b3e2^	0.35 ± 0.04 ^a2b3e2^	18.62	40
400	104.50 ± 5.13^a3c2d1^	6.83 ± 0.70	3.00 ± 0.37^a3c1^	0.34 ± 0.03^a3c3d2^	0.16 ± 0.02^a3c3d2^	30.52	60
CF(mg/kg)							
100	77.50 ± 3.04^b3e3^	9.33 ± 0.84^b1^	6.33 ± 0.71 ^b2^	0.55 ± 0.04^b3e1^	0.37 ± 0.04 ^b3e2^	5.09	15.6
200	86.00 ± 2.86^a2b3e1^	8.67 ± 0.88	5.33 ± 0.71 ^b1^	0.45 ± 0.05^a1^	0.26 ± 0.02^a3^	11.80	28.93
400	105.33 ± 4.65^a3c3d1^	6.83 ± 0.75	3.50 ± 0.56^a2^	0.37 ± 0.01^a3c1^	0.17 ± 0.02^a3c2^	30.52	53.33
NHF(mg/kg)							
100	66.67 ± 2.14^b3^	9.33 ± 1.15	6.50 ± 0.76^b1^	0.58 ± 0.03^b3^	0.40 ± 0.03^b3^	5.09	13.33
200	67.33 ± 2.42^b3^	8.50 ± 1.09	6.33 ± 0.99^b1^	0.52 ± 0.03^b2^	0.40 ± 0.03^b3^	13.53	15.6
400	73.00 ± 2.25^b3^	7.50 ± 1.34	6.17 ± 1.17	0.48 ± 0.04 ^a1 b2^	0.37 ± 0.05^b2^	30.52	17.33

Results are expressed as mean ± SEM (*n* = 6); statistical analysis was performed using one-way ANOVA followed by Tukey’s post hoc test. ^a^Compared to the negative control, ^b^ compared to loperamide, ^c^ compared to 100 mg/kg, ^d^ compared to 200 mg/kg, ^e^ compared to 400 mg/kg. ^1^p < 0.05, ^2^p < 0.01, ^3^p < 0.001.*NC* negative control, *OTD* onset of diarrhea, *TNF* total number of feces, *NWF* number of wet feces, *WTF* weight of total feces, *WWF* weight of wet feces, *ITFO* inhibition of total fecal output, *ID* inhibition of diarrhea

### Castor oil-induced enteropooling

The crude extract at 200 mg/kg and 400 mg/kg significantly decreased both the weight and volume of the intestinal contents (*p* < 0.001) compared to the negative control. Furthermore, 100 mg/kg of the crude extract significantly reduced the weight and volume of the intestinal contents (*p* < 0.01) compared to the negative control. The effect of 400 mg/kg of crude extract was comparable to that of loperamide. Besides, the crude extract at 400 mg/kg significantly reduced the weight and volume of the intestinal content (*p* < 0.001) compared to its 100 mg/kg. A high percentage (75.0%) reduction in intestinal content volume was achieved with loperamide, followed by crude extract (70.83%) at 400 mg/kg. AF at 200 mg/kg and 400 mg/kg significantly reduced intestinal contents weight and volume (*p* < 0.001) compared with negative controls. Furthermore, AF at 400 mg/kg significantly reduced the weight and volume of the intestinal contents compared with its 100 mg/kg and had an effect comparable to that of loperamide. CF at 200 mg/kg (*p* < 0.01) and 400 mg/kg (*p* < 0.001) significantly reduced the weight and volume of intestinal content compared with the negative control. NHF at 400 mg/kg significantly reduced the weight and volume of intestinal contents (*p* < 0.05) compared with the negative control (Table 2).

**Table 2:** Effects of crude extract and solvent fractions of the leaf of *B.abyssinica* on enteropooling

Types of treatment	Weight of intestinal contents (g)	The volume of the intestinal contents (ml)	Reduction of weight of intestinal contents (%)	Reduction of volume of the intestinal contents (%)
NC	0.85 ± 0.04	0.72 ± 0.05	–––––––	–––––––
Loperamide 3 mg/kg	0.26 ± 0.04	0.18 ± 0.04	69.41	75.00
Crude extract (mg/kg)				
100	0.61 ± 0.03^a2b3e3^	0.50 ± 0.05^a2b3e3^	28.24	30.56
200	0.48 ± 0.04^a3b2^	0.37 ± 0.04^a3b1^	43.53	48.61
400	0.33 ± 0.03^a3c3^	0.21 ± 0.03^a3c3^	61.18	70.83
AF(mg/kg)				
100	0.64 ± 0.02 ^a2b3e3^	0.54 ± 0.02^a2b3e2^	24.71	25.00
200	0.50 ± 0.03^a3b3^	0.46 ± 0.03^a3b3^	41.18	36.11
400	0.35 ± 0.03^a3c3^	0.31 ± 0.03^a3c2^	58.82	56.94
CF(mg/kg)				
100	0.67 ± 0.02 ^a1b3^	0.57 ± 0.02 ^b3^	21.18	20.83
200	0.66 ± 0.04^a2b3^	0.55 ± 0.04^a2b3^	22.35	23.61
400	0.53 ± 0.04^a3 b3^	0.42 ± 0.04^a3b2^	37.65	41.67
NHF(mg/kg)				
100	0.81 ± 0.04 ^b3^	0.69 ± 0.05 ^b3^	4.71	4.17
200	0.79 ± 0.03 ^b3^	0.68 ± 0.03 ^b3^	7.06	5.56
400	0.67 ± 0.04 ^a1b3^	0.53 ± 0.04^a1 b3^	21.18	20.83

Results are expressed as mean ± SEM (*n* = 6); statistical analysis was performed using one-way ANOVA followed by Tukey’s post hoc test. ^a^compared to negative control, ^b^compared to loperamide, ^c^compared to 100 mg/kg, ^d^compared to 200 mg/kg, ^e^compared to 400 mg/kg. ^1^p < 0.05, ^2^p < 0.01, ^3^p < 0.001. *NC* negative control

### Antimotility test

The crude extract at 400 mg/kg inhibited the movement of a charcoal meal within the small intestine and decreased the PI significantly (*p* < 0.01) compared to the negative control. Besides, the crude extract at 200 mg/kg inhibited the movement of the charcoal meal within the small intestine and decreased the PI significantly (*p* < 0.05) compared to the negative control. Both 200 mg/kg and 400 mg/kg of the crude extract inhibited the movement of the charcoal meal and decreased the PI comparable to the effect of atropine. The percent inhibition of motility was obtained with the crude extract and its solvent fractions ( Table 3).

**Table 3:** Effects of crude extract and solvent fractions of the leaves of *B. abyssinica* on intestinal motility

Types of treatment	The total length of the intestine (cm)	Length of intestine marked with charcoal (cm)	PI (%)	Inhibition of motility (%)
NEC	57.58 ± 0.87	33.75 ± 1.57	58.76 ± 3.26	–––––––-
Atropine 5 mg/kg	58.50 ± 0.80	18.75 ± 1.57	32.03 ± 2.62	45.49
Crude extract (mg/kg)				
100	59.83 ± 1.72	27.25 ± 1.67^b1^	45.83 ± 3.48^b1^	22.00
200	57.5 ± 0.61	25.17 ± 1.81^a1^	43.92 ± 3.54^a1^	25.26
400	57.73 ± 0.95	23.33 ± 1.76^a2^	40.48 ± 3.17^a2^	31.11
AF (mg/kg)				
100	55.92 ± 0.72	25.67 ± 1.08^a2b1^	45.93 ± 1.97^a1b2^	21.83
200	56.42 ± 1.00	23.33 ± 1.26^a3^	41.43 ± 2.39^a2^	29.49
400	57.00 ± 0.76	22.42 ± 1.34^a3^	39.47 ± 2.82^a3^	32.83
CF (mg/kg)				
100	57.75 ± 1.24	31.33 ± 1.42 ^b3e2^	54.24 ± 2.06^b3e2^	7.69
200	57.92 ± 1.36	28.67 ± 2.00^b2^	49.30 ± 2.67^b3^	16.10
400	57.67 ± 2.46	21.33 ± 1.60^a3c2^	36.89 ± 1.89^a3c2^	37.22
NHF (mg/kg)				
100	57.83 ± 1.07	31.83 ± 1.49 ^b3e2^	55.16 ± 2.86 ^b3e2^	6.13
200	57.17 ± 1.12	30.08 ± 1.65^b3e1^	52.88 ± 3.32^b2e1^	10.01
400	58.08 ± 0.98	21.75 ± 1.53^a3c2d1^	37.67 ± 3.30^a2c2d1^	35.89

Results are expressed as mean ± SEM (*n* = 6); statistical analysis was performed using one-way ANOVA followed by Tukey’s post hoc test. ^a^compared to negative control, ^b^compared to atropine, ^c^compared to 100 mg/kg, ^d^compared to 200 mg/kg, ^e^compared to 400 mg/kg. ^1^p < 0.05, ^2^p < 0.01, ^3p^ < 0.001.*NC* Negative control

The AF at 200 mg/kg and 400 mg/kg inhibited the movement of a charcoal meal within the small intestine significantly (*p* < 0.001) compared to the negative control and had a comparable effect to the effect of atropine. Besides, the AF at 100 mg/kg inhibited the movement of charcoal meal within the small intestine significantly (*p* < 0.01) compared to the negative control. The CF at 400 mg/kg inhibited the movement of a charcoal meal within the small intestine and decreased the PI significantly (*p* < 0.001) compared to the negative control and had a comparable effect to the effect of atropine. In addition, the CF at 400 mg/kg inhibited the movement of the charcoal meal and decreased the PI significantly (*p* < 0.01) compared to its 100 mg/kg. The NHF at 400 mg/kg inhibited the movement of the charcoal meal and decreased the PI significantly compared to the negative control and its 100 and 200 mg/kg.

### *In vivo* antidiarrheal index (ADI)

The ADI of both the crude extract and solvent fractions increased with increasing the dose (Table 4). For instance, ADI of the crude extract increased from 18.06% at 100 mg/kg to 46.91% at 400 mg/kg.

**Table 4:** *In Vivo* ADI of crude extract and solvent fractions of the leaves of *B. abyssinica*

Groups	Dfreq (%)	Gmeq ( %)	Pfreq (%)	ADI (%)
Crude extract (mg/kg)				
100	18.75	22.00	15.60	18.06
200	41.67	25.26	40.00	33.58
400	59.89	31.11	62.27	46.91
AF (mg/kg)				
100	26.30	21.83	13.33	19.13
200	35.94	29.49	40.00	33.65
400	63.28	32.83	60.00	48.04
CF (mg/kg)				
100	20.16	7.69	15.6	13.08
200	33.33	16.10	28.93	24.16
400	63.30	37.22	53.33	48.16
NHF(mg/kg)				
100	3.36	6.13	13.33	6.38
200	4.39	10.01	15.6	8.63
400	13.18	35.89	17.33	19.57
Positive control				
	83.72	45.49	66.67	60.75

*Dfreq* Delay in the onset of diarrhea*, Gmeq* reduction in intestinal transit*, Pfreq* reduction in the number of wet stools

## Discussion

Hydromethanol (80% methanol) can extract non-polar to polar phytochemicals [27]. For this reason, 80% methanol was used as the initial extracting solvent. The in vivo antidiarrheal activity of crude extracts and solvent fractions was evaluated in three models of diarrhea using CO. Ricinoleic acid, the pharmacologically active component of CO binds to and activates the prostanoid EP3 receptor. Ricinoleic acid stimulates the synthesis of endogenous prostaglandins from arachidonic acid in the intestine. Prostaglandins improve gastrointestinal motility, produce a laxative effect, and alter the movement of water and electrolytes in and out of the intestinal lumen [28, 29]. Therefore, the use of CO to induce diarrhea is analogous to the pathophysiology of diarrhea and reasonable to use in this study.

The anti-diarrheal activities of medicinal plants are related to phytochemical constituents including flavonoids, tannins, saponins, sugars, sterols, and/or terpenes [30, 31]. The leaves of *B. abyssinica* contain most of the listed phytochemicals that contribute to its anti-diarrheal activities observed in this study. The mode of action of the antidiarrheal activities of extracts of traditional MPs involves antisecretory activity, favoring intestinal absorption, antispasmodic or antimotility, anti-microbial, and antispasmodic action [32, 33].

After administration of CO at a dose of 0.1–0.3 ml, diarrhea occurs within 1 to 2 h [34]. In this study, CO was administered at a dose of 0.5 ml and negative controls showed diarrhea for an average of 64.5 min. Semipolar secondary metabolites are required to delay the onset of diarrhea [35]. This may justify the observed effect of the crude extract and AF at 200 mg/kg and 400 mg/kg and CF at 400 mg/kg to delay or alleviate diarrhea. Provided n-hexane is non-polar, the lack of activity of NHF at all three doses in delaying the onset of diarrhea may be due to the absence of semipolar secondary metabolites.

Despite the lack of a specific definition of diarrhea, fece weight and consistency are often taken into account [36]. Regarding this, the number of loose or wet feces was recorded in this study. The crude extract, AF, and CF were reduced in the number of wet feces in a dose-dependent manner. A high percentage reduction in the number of wet feces was achieved at 400 mg/kg of crude extract, AF, and CF. The increase in the percentage reduction with increasing doses suggests that high doses are needed to effectively inhibit diarrhea [37].

The antisecretory activity of MPs relies on the presence of phytochemicals including tannins [38] and flavonoids [39]. From previous phytochemical screening studies, tannins and flavonoids of methanol and aqueous extracts and flavonoids (but not tannins) of chloroform extracts were identified as major phytochemical components of the leaf of *B. abyssinian* [3]. Thus the observed activity, in a CO-induced enteroppoling antidiarrheal model, at all three doses of the crude extract and AF and could be due to the presence of tannins and flavonoids in a dose-dependent manner. Similarly, observed antisecretory activity of 200 mg/kg and 400 mg/kg of CF is related to the presence of flavonoids while lack of activity at 100 mg/kg could be due to the absence of tannins and flavonoids. Only 400 mg/kg of NHF showed antisecretory activity may be due to tannins and/or flavonoids being only available at high doses of NHF. A shred of evidence suggests that plant extracts may contain denatured proteins that produce tenant proteins that act as a protective shade for the intestinal lining and reduce secretion [40]. Thus, a decrease in the weight and volume of the intestinal contents in the crude extract and the fractions of the solvent, probably due to the presence of both antisecretory phytochemicals and denatured proteins.

The anti-motility activity of medicinal plants extracts is related to phytochemicals such as tannins, flavonoids, and alkaloids [41]. Based on this the observed antimotility activity at 200 mg/kg and 400 mg/kg of crude extract and all three doses of AF related to the presence of mentioned phytochemicals. As such, the lack of antimotility activity at 100 mg/kg of the crude extract may be related to the absence or low levels of tannins, alkaloids, and flavonoids. In addition, aqueous extract of leaf of *B. abyssinica* was reported to have antispasmodic activity (16) which could contribute to the observed antimotility activity of AF at all three doses. The antimotility activity showed at 400 mg/kg and lack of activity at 100 mg/kg and 200 mg/kg of CF and NHF could be related to the presence and absence or low amount of antimotility phytochemicals, respectively.

ADI is a measure of relative effectiveness for diarrhea inhibition [27]. In this study, ADI was increased in a dose-dependent manner. Although the highest ADI was achieved at 400 mg/kg CF it had the lowest ADI at 100 mg/kg and 200 mg/kg compared with equal doses of the crude extract and AF. This suggests that CF has less antidiarrheal activity at 100 mg/kg and 200 mg/kg.

## Conclusion

The results of this study showed that the crude extract and AF and CF of *B. abyssinica* fresen leaves had significant antidiarrheal activity. This supports the use of the plant in the treatment of diarrhea in traditional medicine, while further studies are required to isolate the specific phytochemicals responsible for the antidiarrheal activity and determine their mechanism of action at the molecular level.

## Data Availability

All the necessary data supporting the result and conclusion have been incorporated into the manuscript.

## Abbreviations

AF: Aqueous fraction
ADI: Antidiarrheal index
CF: Chloroform fraction
CO: Castor oil
MPs: Medicinal plants
NHF: N-hexane fraction
PI: Peristalsis index
